# Ten-Year Real-World Outcomes and Clinicopathologic Predictors of Recurrence in Adult Granulosa Cell Tumors: A Turkish Single-Center Experience †

**DOI:** 10.3390/curroncol32090504

**Published:** 2025-09-10

**Authors:** Aslı Geçgel, Oğuzcan Özcan, Pınar Peker, Gürdeniz Serin, Burçak Karaca Yayla, Erdem Göker, Ulus Ali Şanlı

**Affiliations:** 1 Department of Medical Oncology, Faculty of Medicine, Ege University, Izmir 35100, Türkiye; oguzcan.ozkan@ege.edu.tr (O.Ö.); burcak.karaca.yayla@ege.edu.tr (B.K.Y.); erdem.goker@ege.edu.tr (E.G.); 2 Department of Medical Oncology, Adana City Hospital, Adana 01370, Türkiye; pinar.peker@saglik.gov.tr; 3 Department of Pathology, Faculty of Medicine, Ege University, Izmir 35100, Türkiye; gurdeniz.serin@ege.edu.tr; 4 Department of Medical Oncology, Private Egepol Hospital, Izmir 35270, Türkiye; ulus.ali.sanli@ege.edu.tr

**Keywords:** adult granulosa cell tumor, disease-free survival, prognostic factors, endometrial pathology, adjuvant chemotherapy, advanced stage, long-term outcomes

## Abstract

Adult granulosa cell tumors (AGCT) are rare ovarian neoplasms with typically indolent behavior but potential for late recurrence. This study aimed to evaluate long-term outcomes and identify clinicopathological predictors of disease-free survival (DFS) in patients with AGCTs. This retrospective cohort study included patients with histologically confirmed AGCTs who were treated or followed at Ege University Faculty of Medicine between January 2012 and 2023. Survival outcomes were analyzed using Kaplan–Meier and Cox regression methods. Among 55 patients with a median follow-up of 113.7 months, the median DFS was 92.3 months, and the median overall survival (OS) was 113.7 months. The 5-year DFS and OS rates were 84.5% and 93.9%, respectively. Recurrence occurred in 23.6% of patients and was significantly linked to advanced FIGO stage, atypical endometrial pathology, and bleomycin–etoposide–cisplatin (BEP)/etoposide–cisplatin (EP)-based adjuvant chemotherapy. Larger tumor size (>10 cm) and stage III disease were also associated with shorter DFS. Univariate analysis showed that stage III disease (HR 7.14, *p* = 0.006) and tumor size >10 cm (HR 3.59, *p* = 0.025) were associated with significantly shorter DFS, while absence of endometrial pathology was protective (HR 0.34, *p* = 0.022). In multivariate analysis, stage III disease remained the only independent predictor of recurrence (HR 4.45, *p* = 0.046). Advanced-stage disease is an independent predictor of recurrence and should be considered a high-risk feature requiring prolonged follow-up.

## Simple Summary

Adult granulosa cell tumors (AGCTs) are rare ovarian cancers that usually grow slowly but can recur many years after treatment. Because these tumors are uncommon, predicting which patients are more likely to experience recurrence remains challenging. In this study, we reviewed the records of 55 women who underwent surgery and long-term follow-up at a high-volume cancer center over a ten-year period. We analyzed tumor characteristics, treatments, and outcomes to identify factors that influence prognosis. Most patients were diagnosed at an early stage and had excellent long-term survival; however, advanced-stage disease was associated with a higher risk of recurrence. These findings highlight the importance of expert surgical management, accurate staging, and long-term surveillance in this rare cancer. The study provides real-world data that can guide individualized care decisions and supports future research in refining risk assessment and follow-up strategies for patients with AGCTs.

## 1. Introduction

Adult granulosa cell tumors (AGCTs) are rare ovarian neoplasms of sex cord–stromal origin, representing roughly 2–5% of all ovarian malignancies [1]. Although most cases are identified in peri- or postmenopausal women, they can occasionally occur in younger patients [2].

These tumors generally exhibit indolent behavior, and prognosis is favorable in most early-stage cases. However, their potential for late recurrence (even decades after initial treatment) necessitates prolonged follow-up [2]. While 5-year survival rates often exceed 90% in early-stage disease, relapses significantly compromise long-term outcomes [3]. 

Surgical resection remains the cornerstone of management, although the role of comprehensive staging, including lymphadenectomy, is debated due to the low incidence of nodal metastasis [4]. The benefit of adjuvant chemotherapy is also uncertain, particularly in early-stage disease, as high-quality supporting evidence is lacking. Regimens such as bleomycin–etoposide–cisplatin (BEP), etoposide–cisplatin (EP), and carboplatin–paclitaxel have been used in both adjuvant and recurrent settings; however, their impact on long-term outcomes remains unclear [5].

Molecularly, over 90% of AGCTs harbor the FOXL2 c.402C>G (p.C134W) mutation, which serves as a highly specific diagnostic biomarker; however, its prognostic significance remains uncertain, reinforcing the need to refine risk stratification through clinicopathologic factors [6]. Serum markers such as the anti-Müllerian hormone and inhibin B demonstrate diagnostic utility (particularly for recurrence surveillance) although their prognostic significance is less well defined [7]. Numerous clinicopathological features, including tumor stage, size, mitotic index, residual disease, and endometrial pathology, have been proposed as prognostic indicators [8]. 

Despite numerous retrospective studies, inconsistencies persist regarding the prognostic roles of tumor size, endometrial pathology, and adjuvant chemotherapy, emphasizing the need for updated real-world evidence. This retrospective single-center study assessed clinicopathological characteristics, treatment patterns, and long-term outcomes of AGCT patients, focusing on disease-free survival (DFS) and recurrence risk.

## 2. Methods

This retrospective cohort study included female patients with histopathologically confirmed AGCTs who underwent primary surgery and were subsequently followed at the Department of Medical Oncology, Ege University Faculty of Medicine, between January 2012 and 2023. Clinical and pathological data were obtained from electronic hospital records and patient files. Collected variables included age at diagnosis, menopausal status, parity, presenting symptoms, type of surgery, adjuvant treatment status and regimen, recurrence status, and duration of follow-up. The pathological assessment encompassed measurements of tumor size and location, determination of International Federation of Gynecology and Obstetrics (FIGO) stage, evaluation of mitotic activity, characterization of endometrial pathology, and assessment of estrogen receptor (ER) expression and inhibin status. For disease classification, we applied the 2014 FIGO staging system, which is currently the most widely used and clinically relevant framework for ovarian, fallopian tube, and primary peritoneal malignancies [9]. 

For statistical analysis, certain continuous variables were categorized to facilitate survival comparisons. Age at diagnosis was categorized as ≤65 vs. >65 years; menopausal status was categorized as pre- vs. peri/postmenopausal; tumor size was categorized as ≤10 vs. >10 cm; and mitotic index was categorized as ≤4 vs. >4 mitoses per 10 high-power fields (HPFs), with counts performed in the area showing the highest mitotic activity. Following prior studies, we applied a cutoff of >4 mitoses/10 HPFs to classify tumors into low- and high-mitotic activity groups [10,11,12]. Tumor size was categorized as ≤10 cm or >10 cm based on prior studies demonstrating that tumors larger than 10–15 cm are associated with increased recurrence risk and poorer survival outcomes in AGCT patients [10,13]. This threshold has been used in several retrospective series to stratify patients into clinically meaningful prognostic groups and was therefore adopted for survival analyses in our study.

FIGO stage was classified as early stage (Stage I–II) and advanced stage (Stage III), while parity was categorized as ≤2 and ≥3. Endometrial pathology was classified into three categories: none, non-atypical hyperplasia, and a combined group of atypical hyperplasia/carcinoma. These categorical groupings were used in Kaplan–Meier survival analyses and multivariable regression models.

DFS was calculated from the date of primary surgery to the time of documented recurrence or last disease-free assessment, while OS was measured from surgery to death from any cause or the last recorded follow-up. Patient demographics and clinicopathologic variables were summarized descriptively. Continuous variables, including age, were reported as mean ± standard deviation (SD), as well as median values with interquartile ranges (IQRs), whereas categorical data were expressed as counts and percentages. Survival probabilities were estimated using the Kaplan–Meier method, and subgroup differences were evaluated with the log-rank test. Five-year DFS and OS estimates were derived from Kaplan–Meier survival curves.

The relationships between recurrence status and clinicopathologic parameters were assessed using Pearson’s chi-square test or Fisher’s exact test, as appropriate. Cox proportional hazards regression models were applied for survival analyses, initially in univariate form and subsequently in multivariate models to determine independent predictors of DFS. Variables demonstrating a *p*-value < 0.10 in univariate analyses were entered into the multivariate model. Results are presented as hazard ratios (HRs) with corresponding 95% confidence intervals (CIs) and *p*-values. Forest plots were generated to visually represent the results of the multivariate Cox regression analysis. Due to the limited number of death events (*n* = 5), multivariate regression analysis for overall survival (OS) could not be reliably performed and was therefore omitted.

All statistical analyses were performed using IBM SPSS Statistics for Windows, v22.0 (IBM Corp., Armonk, NY, USA). Statistical significance was defined as a two-sided *p*-value of <0.05. Clinical data were updated before final analysis in July 2025 to ensure the most accurate representation of survival and recurrence outcomes.

This retrospective study received approval from the Ethics Committee of Ege University Faculty of Medicine (Decision No: 25-3.1T/64, 20 March 2025) and was carried out in accordance with the Declaration of Helsinki.

## 3. Results

The study cohort comprised 55 patients with a confirmed diagnosis of AGCT. The mean age at diagnosis was 50.1 ± 14.9 years, with a median of 52 years (IQR: 41–60). Median DFS and OS were 92.3 months and 113.7 months, respectively. Among patients who experienced recurrence, the mean time to relapse was 58.4 months. The estimated 5-year DFS was 84.5% (95% CI: 74.5–94.5%), and the 5-year OS was 93.9% (95% CI: 87.2–100.0%).

More than half of the patients were postmenopausal (52.7%), and 45.5% had no reported comorbidities. Recurrence was observed in 23.6% of patients, most commonly in the peritoneum (10.9%). Regarding associated endometrial pathology, 30.9% had non-atypical endometrial hyperplasia. There was a statistically significant association between menopausal status and endometrial pathology (*p* = 0.002). Among premenopausal patients, seventeen had no endometrial pathology, three had non-atypical hyperplasia, and one had atypical hyperplasia or carcinoma. Among peri/postmenopausal patients, eleven had no endometrial pathology, fourteen had non-atypical hyperplasia, and nine had atypical hyperplasia or carcinoma. ER status was available for 28 patients, while it remained unknown for 27 patients (49.1%). Inhibin expression was positive in 83.6% of cases.

Adjuvant chemotherapy was given to 38.2% of patients, predominantly using BEP/EP regimens. Only one patient (1.8%) underwent a fertility-sparing procedure, while the remaining 92.7% underwent standard primary staging surgery. None of the patients received adjuvant hormonal therapy. Tumors were most frequently located in the left ovary (54.5%) and measured ≤10 cm in 63.6% of cases. Most patients were diagnosed at an early stage (Stage I: 89.1%), and 65.5% had a mitotic index >4 per 10 HPF. The mean follow-up duration was 108.4 months, with a median of 113.7 months. At the time of data cutoff, 90.9% of patients were alive. A detailed distribution of clinicopathological features is provided in Table 1.

Chi-square analyses revealed that recurrence was significantly associated with adverse pathological and clinical features, including atypical endometrial pathology, receipt of BEP/EP-based adjuvant chemotherapy, and Stage III disease (Table 2). Recurrence was more frequent among patients with atypical hyperplasia or carcinoma, those treated with BEP/EP regimens, and patients diagnosed at advanced stage. No significant associations were observed for age, menopausal status, tumor size, mitotic index, Ki-67 index, ER status, or inhibin expression.

**Figure 1:**
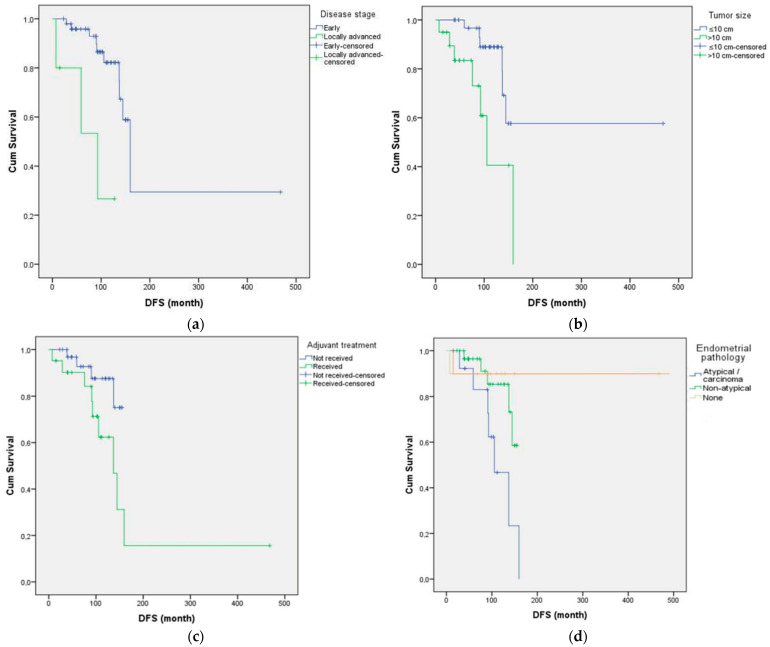
Kaplan–Meier curves for DFS according to key clinicopathological variables. (**a**) DFS by disease stage (early vs. locally advanced), (**b**) DFS by tumor size (≤10 cm vs. >10 cm), and (**c**) DFS by adjuvant chemotherapy status (received vs. not received) (**d**) DFS by endometrial pathology (none, non-atypical, atypical/carcinoma).

Kaplan–Meier analyses demonstrated significantly shorter DFS in patients with tumors >10 cm, advanced-stage disease, and atypical endometrial pathology (log-rank *p* < 0.05; Figure 1). Patients who received adjuvant chemotherapy also showed shorter DFS, likely reflecting selection for higher-risk disease; however, subgroup comparisons between chemotherapy regimens were limited due to small numbers. Other variables, including mitotic index, Ki-67 expression, estrogen receptor (ER) status, tumor laterality, parity, inhibin status, and comorbidity, were not associated with significant differences in DFS (all *p* > 0.05). Figure 1 shows Kaplan–Meier curves for DFS by key prognostic factors: (a) FIGO stage, (b) tumor size, (c) adjuvant chemotherapy status, and (d) endometrial pathology. DFS was shortest in patients with Stage III disease, tumors >10 cm, those receiving adjuvant chemotherapy (reflecting selection for high-risk disease), and patients with atypical endometrial changes, while patients without endometrial pathology had the most favorable outcomes.

Univariate Cox regression analysis identified multiple variables with a significant association to DFS. Patients with Stage III disease demonstrated markedly reduced DFS compared with those in Stage I–II (HR 7.14; 95% CI, 1.78–28.73; *p* = 0.006). Tumor size >10 cm was also associated with worse DFS (HR: 3.59; 95% CI: 1.18–10.95; *p* = 0.025). The absence of endometrial pathology was associated with improved DFS (HR: 0.343; 95% CI: 0.138–0.858; *p* = 0.022). Adjuvant chemotherapy was associated with a nonsignificant trend toward reduced DFS (HR 3.21; 95% CI, 0.96–10.69; *p* = 0.058).

**Figure 2:**
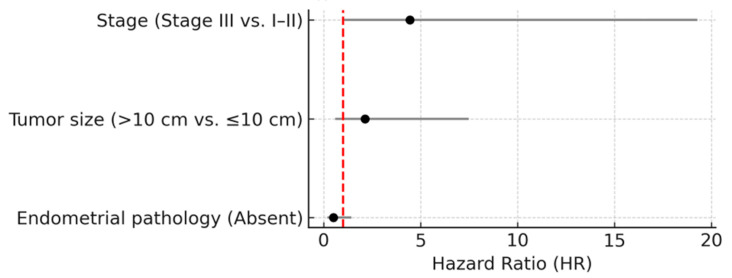
Forest plot summarizing HRs with 95% CIs from univariate and multivariate Cox regression analyses of DFS in AGCT patients. Black bullet points represent HR estimates; solid grey lines indicate 95% CIs. The vertical red dashed line marks HR = 1 (no effect).

In the multivariate Cox regression model, Stage III disease persisted as an independent predictor of reduced DFS (HR 4.45; 95% CI, 1.03–19.27; *p* = 0.046). Tumor size >10 cm (HR: 2.13; *p* = 0.241) and endometrial pathology (HR: 0.51; *p* = 0.197) did not retain statistical significance in the multivariate model. Although adjuvant treatment (BEP/EP or carboplatin–paclitaxel) was included in the multivariate model due to its *p*-value < 0.10 in univariate analysis, it did not demonstrate a statistically significant association with DFS (*p* = 0.537) and was not retained in the final multivariate model. Table 3 summarizes the results of the univariate and multivariate Cox regression analyses for DFS, which are also visualized as a forest plot in Figure 2.

## 4. Discussion

### 4.1. Summary of Main Results

This study provides a detailed analysis of recurrence patterns and DFS in AGCTs. In our cohort of AGCT patients, FIGO stage III disease was an independent predictor of shorter DFS (HR 4.45; 95% CI, 1.03–19.27; *p* = 0.046). While tumor size >10 cm was significant in univariate analysis, it did not retain significance in multivariate models. Although adjuvant chemotherapy showed a significant association with recurrence in the chi-square analysis (*p* = 0.008), this likely reflects confounding by indication, as patients with higher-risk disease features were more likely to receive chemotherapy. After adjusting for stage and tumor size in the multivariate Cox model, this association was no longer statistically significant (HR: 1.28; 95% CI: 0.57–2.85; *p* = 0.537).

The median disease-free and OS were 92.3 and 113.7 months, respectively. The 5-year DFS rate was 84.5% (95% CI: 74.5–94.5%) and the 5-year OS rate was 93.9% (95% CI: 87.2–100.0%). The recurrence rate was 23.6%. Multivariate analysis for OS was not feasible due to the limited number of deaths (*n* = 5).

### 4.2. Comparison with Existing Literature

These findings are consistent with previous reports from multiple large-scale cohorts [14,15]. The Helsinki cohort further supports the unpredictable recurrence pattern of AGCTs reporting a median time of 89 months to relapse and frequent asymptomatic presentations. While tumor rupture was the only independent predictor of recurrence in that study, we identified advanced FIGO stage as the strongest prognostic factor [2]. In our cohort, tumor rupture was documented in only three patients, which precluded meaningful statistical evaluation of its prognostic significance, and this was acknowledged as a limitation of the study. Multiple retrospective studies have confirmed that advanced FIGO stage (beyond IA) is associated with shorter DFS and OS in AGCTs, underscoring the prognostic importance of tumor stage at diagnosis across different populations [16,17,18]. 

In a study of 61 patients with AGCTs, the recurrence rate was reported as 26%, with a median relapse interval of 5.5 years. The 5-year DFS and OS rates were 84% and 93%, respectively [19], which are comparable to our results of 84.5% and 93.9%. Similarly, Şahin et al. reported 5-year disease-free and OS rates of 85% and 100%, respectively, with a lower recurrence rate (13.4%) than in our series (23.6%). Advanced FIGO stage and positive peritoneal cytology were independent predictors of shorter DFS [20]. Supporting prolonged follow-up, recent studies show that up to one-third of recurrences occur after 10 years, especially in stage IC and cyst rupture cases [21]. In contrast, a small retrospective study of 18 patients found no significant difference in DFS across FIGO stages (*p* = 0.52), likely due to early-stage predominance and low event rate [22]. Another multicenter prospective study of 208 patients reported no benefit of additional staging surgery and suggested that follow-up beyond five years may be unnecessary, as survival did not differ between recurrences detected during scheduled visits and those diagnosed symptomatically [23]. Although OS analysis was limited in our cohort due to the low number of observed death events (*n* = 5), the 5-year OS rate of 89.3% closely aligns with previously reported ranges in the literature (85–95%). Large retrospective series consistently report favorable survival outcomes in early-stage AGCTs: OS exceeding 90% has been described for early-stage cases in institutional studies [20,24,25,26]. Collectively, these data reinforce the indolent but persistent nature of AGCTs, underscore the generally favorable long-term prognosis when diagnosed early, and support our observed OS despite incomplete multivariate modeling.

Stage III disease was independently associated with shorter DFS in our cohort, consistent with the MITO study, which emphasized the risk of late recurrence. While the MITO study reported a median recurrence time of 53 months and 47% of relapses beyond five years, our cohort showed a similar interval but a lower five-year recurrence rate (32.7%) [3]. This lower rate may be attributed to the predominance of early-stage diagnoses and standardized surgical management at a high-volume tertiary center, emphasizing the value of expert care. With a median follow-up of nearly 10 years, the mean time to recurrence was 58.4 months, and several relapses occurred beyond five years, underscoring the indolent but late-relapsing nature of AGCTs. These findings support a risk-adapted follow-up strategy, particularly for patients with advanced stage.

Consistent with the national Turkish TOG cohort, our study demonstrated comparable 5-year DFS (84.5% vs. 86%) and confirmed advanced FIGO stage as a key predictor of recurrence. Both cohorts also linked atypical endometrial pathology and postmenopausal status to poorer outcomes, suggesting hormone-dependent mechanisms supported by the estrogen-secreting nature of AGCTs. In the TOG cohort, hyperplasia and concurrent endometrial carcinoma were observed in 30% and 7.5% of cases, respectively [8]; similarly, our cohort showed rates of 12.7% and 5.4%, predominantly in postmenopausal women (*p* = 0.002). Similarly, a large retrospective analysis in Japan reported complex atypical hyperplasia in 7.3% and low-grade endometrial carcinoma in 3.1% of cases, with tumor size significantly predicting synchronous endometrial cancer in menopausal patients [27]. These findings underscore the need for thorough endometrial assessment, especially in patients over 40 or those with abnormal uterine bleeding. Although ER status was unknown in many cases, prior studies suggested a possible association with hormone responsiveness, though its prognostic significance remains uncertain.

In our study, tumor size >10 cm was associated with shorter DFS in univariate analysis. Consistent with our findings, several retrospective and multicenter studies have shown that although larger tumor size may initially appear prognostic, it often loses independent significance after adjusting for confounding factors such as stage and completeness of surgery [13,28,29]. These patterns collectively suggest that larger tumor size may reflect increased tumor burden or surgical complexity rather than being a direct driver of recurrence. Standardizing tumor size thresholds and reporting criteria in future studies is essential to clarify its true prognostic role.

Consistent with our findings, multiple large-scale analyses have consistently reported no significant benefit of adjuvant chemotherapy on recurrence or survival outcomes in AGCTs, even in advanced or recurrent stages. Some studies have even associated adjuvant treatment with worse progression-free or DFS, raising concerns about potential overtreatment in low-risk patients [5,20,30,31]. Notably, the MITO-9 study confirmed that adjuvant chemotherapy lacked independent prognostic significance in FIGO stage IC patients, whereas incomplete surgical staging and non-specialized care settings were stronger predictors of recurrence [32]. In our cohort, shorter DFS observed in chemotherapy-treated patients is most likely attributable to treatment selection bias, as those with higher-risk clinical features were preferentially selected for adjuvant therapy. This reinforces the notion that chemotherapeutic regimens may not improve prognosis unless applied in clearly defined high-risk cases. Given the lack of randomized data, clinicians should be cautious when generalizing the potential benefits of adjuvant chemotherapy. Consistent with previous studies, our findings do not support its routine use in AGCTs; instead, treatment decisions should be individualized within multidisciplinary care pathways, preferably in specialized centers.

Although FOXL2 mutation is present in over 90% of AGCTs and serves as a highly specific diagnostic marker, molecular analysis could not be performed in our study. Given emerging evidence suggesting a potential prognostic role for FOXL2 and other molecular alterations [33,34], future multicenter studies integrating comprehensive genomic profiling are warranted to better define molecular risk factors and guide individualized management strategies.

### 4.3. Strengths and Weaknesses

This study provides one of the most up-to-date analyses of AGCTs, incorporating clinical follow-up data as of July 2025. The inclusion of late recurrences (a hallmark of AGCTs) enhances the accuracy of long-term outcome assessment. A key strength is consistency in diagnostic, surgical, and therapeutic management, as all patients were treated at a single tertiary center, reducing inter-institutional variability and strengthening internal validity. The study offers detailed clinicopathological data, including tumor size, mitotic index, disease stage, endometrial pathology, ER status, and adjuvant treatment.

We employed rigorous statistical approaches, utilizing Kaplan–Meier survival estimates, log-rank comparisons, and Cox proportional hazards modeling based on clinically meaningful cutoffs. Analytical transparency was maintained by addressing discrepancies between mean DFS and Kaplan–Meier estimates, likely due to censoring and subgroup imbalances. This study offers real-world evidence on adjuvant chemotherapy practices in AGCTs, an area with limited prospective research, and further examines the timing and patterns of disease recurrence.

However, several limitations should be acknowledged. The retrospective design may introduce selection and information biases. A formal sample size calculation was not performed given the rarity of AGCT, and the small cohort size limits statistical power and increases the risk of type II error, particularly in multivariate analyses. The absence of FOXL2 mutational testing, a well-established diagnostic marker with limited prognostic data, prevented exploration of potential molecular outcome associations. Nearly half of the cohort (49.1%) had unknown ER status, which restricts interpretation of its prognostic relevance. Due to limited subgroup sizes, statistical comparisons between adjuvant chemotherapy subgroups were not feasible. The paradox of longer mean DFS despite early recurrences in certain subgroups likely reflects censoring effects and heterogeneity. Finally, serum anti-Müllerian hormone levels were inconsistently available, preventing evaluation of their prognostic or surveillance value.

These limitations underscore the need for larger, prospective multicenter studies with standardized molecular profiling to validate biomarkers and guide individualized, risk-adapted strategies in AGCTs.

## 5. Conclusions

Taken together, our findings highlight the importance of individualized risk stratification and prolonged surveillance in patients with AGCTs, particularly those with advanced-stage disease. The elevated recurrence risk observed in stage III cases may reflect microscopic peritoneal dissemination or suboptimal cytoreduction, thereby justifying follow-up beyond the conventional 5-year period. These results underscore the value of clinicopathology-based decision-making and advocate multicenter collaborations to enhance prognostic modeling and optimize management in this rare tumor entity.

Future prospective studies integrating centralized pathology review and molecular profiling are warranted to validate these observations. Incorporating emerging biomarkers, such as FOXL2 mutation status or circulating tumor DNA, may also enable risk-adapted follow-up strategies and facilitate earlier detection of recurrence.

## Figures and Tables

**Table 1:** Distribution of clinical and pathological features.

Variable	Category	*n* (%)
Menstrual Status	Premenopausal	21 (38.2%)
	Perimenopausal	5 (9.1%)
	Postmenopausal	29 (52.7%)
Comorbidity Count	None	25 (45.5%)
	One	11 (20.0%)
	≥2	19 (34.5%)
Recurrence	Absent	42 (76.4%)
	Present	13 (23.6%)
Recurrence Site	Local recurrence	4 (7.3%)
	Visceral metastasis	3 (5.5%)
	Peritoneal	6 (10.9%)
Endometrial Pathology	None	28 (50.9%)
	Non-atypical hyperplasia	17 (30.9%)
	Atypical hyperplasia	7 (12.7%)
	Carcinoma	3 (5.4%)
Estrogen Receptor Status	Positive	18 (32.7%)
	Negative	10 (18.2%)
	Unknown	27 (49.1%)
Inhibin Status	Negative	9 (16.4%)
	Positive	46 (83.6%)
Adjuvant Treatment	None	34 (61.8%)
	BEP/EP	16 (29.1%)
	Carboplatin–paclitaxel	5 (9.1%)
Parity	Unknown	6 (10.9%)
	≤2	33 (60.0%)
	≥3	16 (29.1%)
Symptoms	Menstrual irregularity	8 (14.5%)
	Abdominal distension	21 (38.2%)
	Postmenopausal bleeding	18 (32.7%)
	Vaginal bleeding	5 (9.1%)
	Acute abdomen	1 (1.8%)
	Incidental	2 (3.6%)
Surgery Type	Primary staging	51 (92.7%)
	Other	4 (7.2%)
Tumor Size	≤10 cm	35 (63.6%)
	>10 cm	20 (36.4%)
Tumor Location	Right	25 (45.5%)
	Left	30 (54.5%)
Pathological Stage	Stage 1	49 (89.1%)
	Stage 2	1 (1.8%)
	Stage 3	5 (9.1%)
Mitotic Index	≤4	19 (34.5%)
	>4	36 (65.5%)
Vital Status	Alive	50 (90.9%)
	Deceased	5 (9.1%)

**Table 2:** Association between clinicopathological factors and recurrence status (chi-square test). *p* < 0.05 was considered statistically significant.

Variable	Category Comparison	Chi-Square (χ^2^)	*p*-Value
Endometrial pathology	None vs. Non-atypical vs. Atypical/Carcinoma	7.389	0.025
Adjuvant chemotherapy	No vs. BEP/EP or Carboplatin–Paclitaxel	6.953	0.008
Stage	Stage I–II vs. Stage III	4.029	0.045
Age group	≤65 vs. >65	0.101	0.751
Menopausal status	Pre vs Peri/Post	0.458	0.498
Tumor size	≤10 cm vs. >10 cm	2.248	0.134
Mitotic index	≤4 vs. >4	0.99	0.32
Ki-67 index	≤10% vs. >10%	0.103	0.749
Estrogen receptor status	Negative vs. Positive vs Unknown	0.408	0.815
Inhibin expression	Negative vs Positive	0.561	0.454

**Table 3:** Univariate Cox regression analysis for DFS. *p* < 0.05 was considered statistically significant.

Variable	Univariate HR (95% CI)	*p*-Value	Multivariate HR (95% CI)	*p*-Value
Stage (Stage III vs. I–II)	7.14 (1.78–28.73)	0.006	4.45 (1.03–19.27)	0.046
Tumor size (>10 cm vs. ≤10 cm)	3.59 (1.18–10.95)	0.025	2.13 (0.60–7.49)	0.241
Endometrial pathology (Absent)	0.343 (0.138–0.858)	0.022	0.51 (0.18–1.42)	0.197
Adjuvant chemotherapy (Yes vs. No)	3.21 (0.96–10.69)	0.058	-	-
Mitotic index (>4 vs. ≤4)	1.40 (0.54–3.63)	0.488	-	-
Menopausal status	0.866 (0.501–1.495)	0.605	-	-
Ki-67 (>10% vs. ≤10%)	1.01 (0.40–2.58)	0.984	-	-
Estrogen receptor positivity (Yes vs. No)	1.17 (0.45–3.06)	0.746	-	-
Tumor laterality (Unilateral vs. Bilateral)	1.23 (0.47–3.25)	0.675	-	-
Parity (≥3 vs. ≤2)	0.85 (0.33–2.21)	0.740	-	-
Inhibin positivity (Yes vs. No)	0.77 (0.27–2.23)	0.620	-	-
Comorbidity (Yes vs. No)	1.11 (0.43–2.86)	0.820	-	-

## Data Availability

The datasets used and/or analyzed during the current study are available from the corresponding author upon reasonable request.
